# TSpred: a web server for the rational design of temperature-sensitive mutants

**DOI:** 10.1093/nar/gku319

**Published:** 2014-04-29

**Authors:** Kuan Pern Tan, Shruti Khare, Raghavan Varadarajan, Mallur Srivatsan Madhusudhan

**Affiliations:** 1 Bioinformatics Institute, 30 Biopolis Street, #07-01, Matrix, Singapore 138671; 2 School of Computer Engineering, Nanyang Technological University, Singapore 639798; 3 Molecular Biophysics Unit, Indian Institute of Science, Bangalore 560012, India; 4 Indian Institute of Science Education and Research, Pune 411008, India; 5 Department of Biological Sciences, National University of Singapore, Singapore 117543

## Abstract

Temperature sensitive (Ts) mutants of proteins provide experimentalists with a powerful and reversible way of conditionally expressing genes. The technique has been widely used in determining the role of gene and gene products in several cellular processes. Traditionally, Ts mutants are generated by random mutagenesis and then selected though laborious large-scale screening. Our web server, TSpred (http://mspc.bii.a-star.edu.sg/TSpred/), now enables users to rationally design Ts mutants for their proteins of interest. TSpred uses hydrophobicity and hydrophobic moment, deduced from primary sequence and residue depth, inferred from 3D structures to predict/identify buried hydrophobic residues. Mutating these residues leads to the creation of Ts mutants. Our method has been experimentally validated in 36 positions in six different proteins. It is an attractive proposition for Ts mutant engineering as it proposes a small number of mutations and with high precision. The accompanying web server is simple and intuitive to use and can handle proteins and protein complexes of different sizes.

## INTRODUCTION

Temperature sensitive (Ts) mutants of a protein are those whose levels of activity decrease when temperature rises above a certain restrictive temperature. Below the restrictive temperature, in permissive temperatures, the mutants and the wild type protein have similar activity levels. Ts mutants are powerful tools to study protein function *in vivo* and in cell culture (1,2). They provide a reversible mechanism to lower the level of a protein simply by changing the temperature of growth (3). There are several practical applications of engineering Ts mutants. In developmental biology, such mutants would provide valuable insight into the functioning of essential genes and those used in multiple phases of development. Ts mutants have also been utilized in many to investigate protein folding pathways (4), macro-molecular assembly (5), controlling the genotype of a cell *in vivo* (6), nervous system defects (7), phenotypic effects (8,9), coordination between different genes (9) pinpointing the phase at which genes are functioning during the cell-division cycle (10), controlled cell-arrest and synchronization of cells or gene functions by means of reversible cell arrest (11,12) and conditional expression of genes in *Drosophila* (13,14).

Ts mutants possess several advantages over other methods such as CRISPER and RNAi (15,16) in producing conditional expression/induction/repression of genes. The advantages include fast temporal response, high reversibility and the applicability to any tissue type or developmental stage of an organism. Various strategies have been proposed to construct Ts mutants, such as by fusion to a heat-sensitive degron (17–19) or insertion of a Ts intein (20). However, Ts mutants are still most commonly generated by random mutagenesis. The process involves using chemical mutagens, ultraviolet (UV) radiation or error prone Polymerase chain reaction (PCR) techniques to introduce random mutations to protein-coding deoxyribonucleic acid (DNA). A screening procedure is then adopted to select Ts mutants (7,21). This procedure is typically laborious and expensive, as a large number of mutants need to be screened. For instance, identifying Ts mutants in the fruit fly *Drosophila melanogaster* involves the screening of several hundreds of thousands of progeny (7). This method is not feasible for model organisms with long generation times, and where it is impractical to obtain large numbers of progeny (22).

To overcome the difficulties posed by random mutagenesis, we have previously demonstrated that it is possible to accurately predict, purely from protein sequence, a small subset of candidate positions, that when mutated, are likely to result in a Ts mutant (13,14,22,23). The method is based on the observation that mutations at buried residue positions can cause large changes in protein thermal stability. Further, the likelihood of an amino acid residing at a buried position can be inferred from its hydrophobicity and that of its flanking residues. Two parameters, namely the average hydrophobicity (24,25) and hydrophobic moment (26) were computed to estimate this likelihood. Following prediction of buried positions, substitutions were suggested at such positions to generate a Ts phenotype. The mechanisms responsible for temperature sensitive phenotype for different proteins could be case-specific and is dependent on complex factors, such as the rate of protein synthesis, susceptibility to proteolysis and whether chaperones are involved in the folding or degradation of the protein. Instead of speculating on the exact mechanism, our approach is to suggest mutations with a set of stereochemically diverse amino acids. It is assumed that these mutations will destabilize the protein to different extents, and that at least one mutant is likely to be temperature sensitive. Through large-scale analysis of known Ts mutants, prediction rules were generated based on the two parameters described above (22). This strategy for choosing mutant substitutions has been experimentally tested previously on CcdB, TBP (TATA binding protein), T4 lysozyme and Gal4 (13,23).

In addition to the sequence-based method described above, the present study enhances Ts mutant prediction by incorporating structural information of the protein. This structural information can be inferred from existing protein structures in the Protein Data Bank (PDB) or from homology models. To determine the degree of burial of an amino acid in a protein, the residue depth measure was used. Depth is defined as the distance of any atom/residue to the closest bulk water (27). It has been shown to accurately measure burial and parameterizes the local protein environment (28,29). Importantly, the depth measure correlates well with structural stability (27) and free energy change of cavity-creating mutations in globular proteins (27,29).

The aim of this study is to predict, a small set of residues that when appropriately mutated, result in temperature sensitive mutants of a given protein. Our server reports the predictions by both the sequence- and structure-based methods. These mutants can be readily tested experimentally. It should be noted that no attempt is made to identify all possible Ts mutants.

In the sections below we first describe the methods used for sequence- and structure-based predictions. This is followed by a description of the benchmark results. The server functionality is then described and illustrated with a case study.

## MATERIALS AND METHODS

In designing Ts mutants we have made use of the observation that the Ts phenotype correlates with decreased protein stability (30,31). In general, this reduction in stability, and hence protein activity, can be attributed to the loss of thermal stabilization contributed by hydrophobic residues in the protein core. Accordingly, numerous experimental studies have shown that significant destabilization in protein stability and activity can be achieved by mutating buried residues as compared to mutating residues at the surface (32–34). Our strategy to predict Ts mutant positions is to target buried positions occupied by hydrophobic residues. We identify such positions using both sequence and 3D structural information.

### Prediction based on primary sequence

The details of the sequence-based predictions have been described at length in an earlier study (22). Qualitatively, the method was based on the observations that the seven residues, Cys, Phe, Ile, Val, Trp, Met and Leu were observed to have an average side chain solvent accessible area of less than 20%. Cysteines are not considered, as they could also be involved with disulphide bond formation or metal ion coordination. The other six residues are mutation targets if predicted as buried. Burial predictions were made using the local average Rose hydrophobicity (22,35) and the hydrophobic moment of the target residue and its flanking neighbours. A set of rules based on these values, predicted degree of residue burial.

### Prediction based on 3D structure

3D structural information would make it easier to identify hydrophobic residue positions that are buried in the core of the protein. We estimate degree of burial by the depth measure (27–29). The depth of a residue (or atom) measures its distance to the closest bulk molecule of bulk solvent (water). In earlier studies we have made a convincing case of how depth gives a more stratified description of residue burial/environment (27) than the widely used solvent accessibility measure.

The average depths of amino acid residues that correspond to an average of 5% side chain accessibility were determined from a non-redundant dataset of 561 proteins extracted from the PDB (36). This dataset was non-redundant to 30% in sequence identity and consisted of single domain (chain length of 120–180 amino acids), high-resolution (resolution ≤ 1.7 Å, R-free ≤ 0.2) protein structures in the PDB (http://mspc.bii.a-star.edu.sg/TSpred/supplementary_data.html). The threshold depth values of Val, Ile, Leu, Met, Phe and Trp were determined to be 6.25, 6.75, 6.75, 7.00, 7.00 and 7.00 Å, respectively. Residues with depths greater than their respective threshold would be predicted as Ts mutant positions. Note, the 5% accessibility cut off value was determined in an earlier study (23) as an optimal value to define buried residues.

### Homology modeling protocol

When no experimentally determined structure is available for a protein sequence of interest (query sequence), a homology model is constructed. Residue burial is then inferred from the 3D model. The TSpred server makes use of an automated homology modeling pipeline that includes steps for template selection, target-template alignment, structure modeling and model assessment. Suitable structural templates are searched in the PDB database using three iterations of PSI-BLAST (version 2.2.28) (37) utilizing the BLOSUM62 substitution matrix (38). A stringent *e*-value cut-off of 0.0001 was used to identify homologs. Among the hits, the one with the best *e*-value is chosen as template. Next, a sequence alignment between query sequence and the template sequence was constructed using SALIGN (39). The resulting alignment is input to the automodel protocol of MODELLER (40) to construct a 3D model of the protein. The model is assessed for accuracy/reliability with the GA341 (41,42) and Discrete Optimized Protein Energy (DOPE) statistical potentials (43). Residue burial information is only taken from those models that satisfy a stringent GA341 and DOPE cutoff of 0.75 and −1, respectively. When no suitable templates are available or the constructed models do not satisfy the GA341 and DOPE criteria, the burial prediction is made using the sequence-based method alone.

### Suggested mutations

At the predicted positions, we suggest that the original residue be mutated to Ala, Trp, Asn, Asp or Pro. While the current program does not rank order amongst these mutations, the suggestion is that one or more of them are likely to result in a Ts phenotype.

### Benchmark datasets

We benchmarked our method by examining the agreement between our predictions with experimentally validated Ts mutants. Thirty six mutants from a set of six proteins, for which extensive mutagenesis data exists, constituted our benchmark. The proteins were gene V (PDB: 1YHA), lambda repressor (PDB: 1LMB), T4 Lysozyme (PDB: 2LZM), CcdB (PDB: 3VUB), Gal4 (PDB: 3CQQ) and Ura3 (PDB: 1DQW)) (13,23,44–49). The performance of our predictions was assessed by coverage (the number of predictions) and precision (number of true positives / number of predictions). Results from both sequence-based and structure-based methods are reported.

For a query sequence without a PDB entry, structural information was inferred from a homology model. The accuracy of the model is largely determined by sequence identity to the structural template used. To gauge the effect of template sequence identity on Ts mutant prediction performance, we built models of T4 lysozyme using 15 templates of varying sequence similarity. The templates were identified by searching DBAli (http://www.salilab.org/DBAli/) (50) for structures similar to T4 lysozyme (PDB 2LZM). We defined similarity as a minimum MAMMOTH *P*-value of 10 and at least 100 equivalent positions (61% structure overlap with T4 lysozyme). The selected templates were between 22 and 91% identical in sequence to T4 lysozyme and all identifiable by PSI-BLAST search with an *e*-value cutoff of 0.0001.

## RESULTS

### Performance on experimentally validated benchmark

Our methods successfully predicted all 36 experimentally validated Ts mutant positions in the six proteins, CcdB, Lysozyme, gene V, gal4, Ura3 and Lamda repressor (Table 1). The sequence-based method correctly predicted 22 and the structure-based method 28 of the 36 cases. The two methods were correct together in 14 of the cases. In addition to the correct predictions, the sequence-based method made one confirmed false positive identification, Val53 in CcdB. Our methods also made 71 other predictions in these six proteins that are yet to be experimentally validated (http://mspc.bii.a-star.edu.sg/TSpred/supplementary_data.html).

**Table 1.** Ts mutant position as predicted by sequence-based, structured-based or both methods

Protein	PDB ID	Chain length	Residue position	Residue type	Prediction method
gene V	1YHA	87	35	VAL	Both
			45	VAL	Both
			47	ILE	Structure
			63	VAL	Structure
			81	LEU	Structure
			78	ILE	Sequence
lambda repressor	1LMB	92	51	PHE	Both
			65	LEU	Both
			76	PHE	Both
			84	ILE	Both
			18	LEU	Structure
			36	VAL	Structure
			47	VAL	Structure
T4 lysozyme	2LZM	164	6	MET	Both
			102	MET	Both
			149	VAL	Structure
			153	PHE	Structure
			103	VAL	Sequence
CcdB	3VUB	101	17	PHE	Both
			18	VAL	Both
			33	VAL	Both
			34	ILE	Both
			54	VAL	Both
			5	VAL	Structure
			36	LEU	Structure
			63	MET	Structure
			50	LEU	Sequence
			53*	VAL	Sequence
			96	LEU	Sequence
			97	MET	Sequence
			98	PHE	Sequence
Gal4	3COQ	88	68	PHE	Both
			69	LEU	Sequence
			70	LEU	Sequence
Ura3	1DQW	267	25	MET	Structure
			32	LEU	Structure
			118	ILE	Structure

The wild-type residue (three-letter amino acid code) at the position is listed under residue type. * The prediction of VAL53 in CcdB as a Ts mutant position is a false positive identification.

The amino acids that we target for mutation—Val, Ile, Leu, Met, Phe and Trp constitute 29.2% (544 residue positions) of the amino acids in these six proteins. Only 19.8% (108 residue positions) of these are identified by our predictions as potential Ts mutant positions. The sequence based method accurately identifies 53% (57 positions) and the structure-based method identifies 79% (85 positions), while 32% (34 positions) is common to both the methods.

### Effect of model accuracy on prediction

In the case of T4 lysozyme, 15 homology models were built using templates with sequence identity ranging from 22 to 91%. The aim was to determine the efficacy of structure-based prediction with homology models of varying accuracy. Out of five experimentally validated Ts mutant positions, high accuracy models (template sequence identity >40%) correctly identified four mutant positions (Table 2). For models built with low target-template sequence identity (<25%) templates, their DOPE and GA341 scores failed to cross the acceptance threshold. These models only predict one or two of the validated positions. The sequence-based method, being independent of the model accuracy, predicted three positions correctly in each of the cases. Note, for both the structure- and sequence-based methods many other positions are predicted by our program. As these positions have not been validated or invalidated as Ts mutation sites, these are not considered as false positives of our method.

**Table 2.** Ts mutant prediction in T4 lysozyme when homology models (identified by their templates) of different accuracies are used

Template quality			Number of predictions				Experimentally validated mutant positions				
PDB:chain	Sequence ID (%)	DOPE	GA341	Sequence	Structure	Both	M6	M102	V103	V149	F153
1pqj:A	90.8	−2.08	1.00	3 (8)	4 (11)	2 (3)	Both	Both	Sequence	Structure	Structure
1d3n:A	86.1	−1.96	1.00	3 (8)	4 (11)	2 (3)	Both	Both	Sequence	Structure	Structure
1t8a:A	81.6	−1.65	1.00	3 (8)	4 (13)	2 (3)	Both	Both	Sequence	Structure	Structure
1cx6:A	79.9	−2.03	1.00	3 (8)	4 (11)	2 (3)	Both	Both	Sequence	Structure	Structure
1lpy:A	78.8	−1.95	1.00	3 (8)	4 (10)	2 (3)	Both	Both	Sequence	Structure	Structure
1swz:A	77.5	−2.21	1.00	3 (8)	4 (13)	2 (3)	Both	Both	Sequence	Structure	Structure
1lwk:A	77.0	−1.74	1.00	3 (8)	4 (12)	2 (3)	Both	Both	Sequence	Structure	Structure
1swy:A	74.5	−2.22	1.00	3 (8)	4 (12)	2 (3)	Both	Both	Sequence	Structure	Structure
1sx2:A	72.4	−2.28	1.00	3 (8)	4 (13)	2 (4)	Both	Both	Sequence	Structure	Structure
1wth:A	43.2	−1.49	1.00	3 (8)	3 (12)	1 (2)	Sequence	Both	Sequence	Structure	Structure
1k28:A	43.2	−1.43	1.00	3 (8)	5 (15)	3 (5)	Both	Both	Both	Structure	Structure
2anv:A	24.2	0.54	0.12	3 (8)	1 (11)	1 (2)	Sequence	Both	Sequence		
2anx:B	23.9	0.60	0.08	3 (8)	2 (11)	2 (3)	Sequence	Both	Both		
2anv:B	23.5	0.49	0.13	3 (8)	2 (11)	2 (3)	Sequence	Both	Both		
2anx:A	22.1	0.64	0.13	3 (8)	1 (10)	1 (2)	Sequence	Both	Sequence		

The number of predictions made by the sequence-based, structure-based or both methods are listed for each of the models with the number of experimentally validated predictions within brackets. The performance of the different models on the experimentally validated mutant positions are additionally shown in separate columns.

### Server description

Our server supports both sequence and structure inputs, and several options are provided for each. For input sequences, users could either specify a database, GenBank (51) or UniProt (52,53), identification number or upload the protein amino acid sequence in FASTA format. If the input consists of multiple sequences, Ts mutant predictions are made for each of the sequences separately. For input structures, the users could either specify the four-letter PDB code with optional additional letters to select specific chains of the protein (the biological unit is used for predictions) or upload a file in PDB format. User uploaded structures are used for prediction without any model assessment.

Our server uses sequence and structure information to make Ts mutant position predictions. The web server outputs the residue positions that have been predicted as Ts mutation targets and prediction method (structure-based, sequence-based or both). In the case where a homology model was used, the server only displays the results of the structure-based method if the models satisfy the assessment criteria.

The user has the option to review details of the hydrophobic moment, average hydrophobicity and residues flanking of predicted mutant positions. In cases where 3D structural data have been used in making the Ts mutant predictions, the PDB structure or the homology model is displayed using a Jmol plugin (http://www.jmol.org/). The backbone chain trace of the proteins is coloured according to the residue depth and the mutant positions are shown as spheres centered on the C^α^ atoms. The spheres are also coloured according to residue depth. In the case of homology models, the user is notified about the target-template sequence identity and the alignment used in model construction is provided.

### Server availability

The server is freely accessible at http://mspc.bii.a-star.edu.sg/TSpred and has no login requirements.

### Case study

We demonstrate the functioning of our server using *Escherichia coli* CcdB protein as an example. CcdB is a poison of DNA gyrase and is a potent cytotoxin (10,11). CcdB is 101 amino acid residues long and its functional form (biological unit) is a homodimer. CcdB was chosen because saturation mutagenesis experiments have been performed (23) on this protein and can be used for validation.

**Figure 1.**
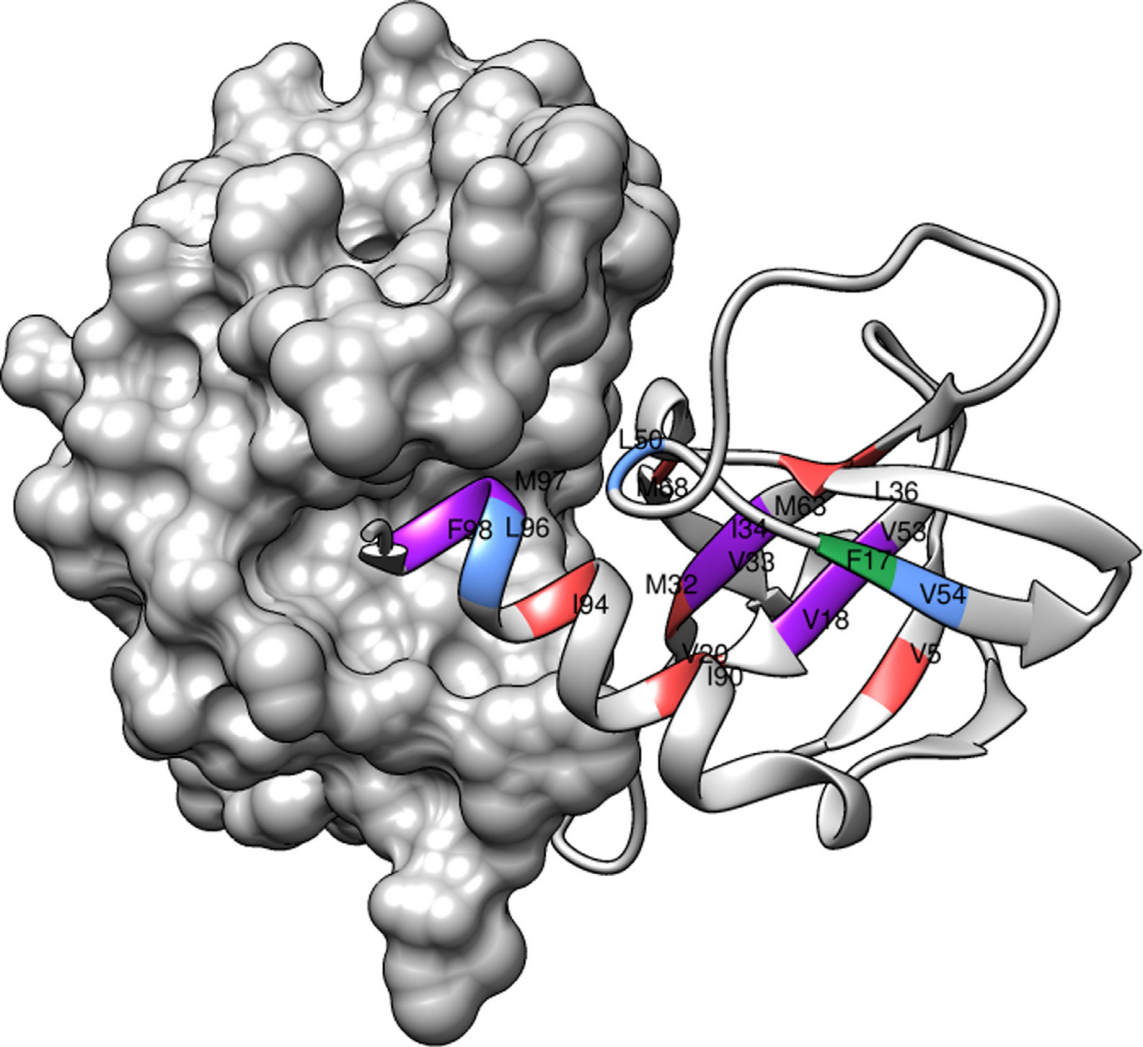
A functionally active CcdB dimer with one monomer in surface representation and the other in ribbon representation. The labeled location of the predictions made by the sequence-based, structure-based and both predictions are coloured blue, red and purple, respectively. The false positive identification is shown in green. The figure was rendered using Chimera (54).

The sequence-based method identified 10 target positions (F17, V18, V33, L34, L50, V53, V54, L96, M97, F98) and the structure-based method identified 14 target positions (V5, F17, V18, V20, M32, V33, L34, L36, M63, M68, I90, I94, M97, F98) (Figure 1). Eight Ts mutant targets (V5, V20, M32, L36, M63, M68, I90, I94) were exclusively predicted by the structure-based method. All the predictions were experimentally verified (100% accuracy). Four target positions were exclusively predicted by the sequence-based method, viz. L50, V53, V54 and L96. Of these, V53 is a false positive. V54 (depth 6.18 Å) only marginally missed the depth threshold (6.25 Å) for structure prediction; L50 and L96 were not buried but their side chains were within 4 Å of active site residues. It was proposed that these residues were important in maintaining the conformation of active site residues, and were hence potential Ts mutant positions. The sequence- and structure-based methods when applied independently, predict ∼10–15% of all residues to be potential Ts mutant positions, corresponding to 50–75 mutation suggestions (five mutations at each position). There are six predictions common to both methods—F17, V18, V33, L34, M97 and F98. All these residues were experimentally verified Ts mutants (23). The structure-based method helps reduce the false positives from sequence-based method and reduces the number of predictions to 6% of all residues or equivalently ∼30 mutant suggestions in CcdB.

We also compared the coverage between sequence- and structure-based predictions. The saturation mutagenesis data shows that CcdB consists of 55 possible Ts positions (23). As our algorithm aims primarily to predict buried hydrophobic residues as Ts mutants, active site (I24, I25, D26, E87, N88, K91, N92, N95, W99, G100, I101), dimerization site (Q2, V20, Q21, S22, I25, T27, M32, T66, M68, A69, I94, N95, M97, F98, W99, G100) and non-hydrophobic residues were excluded from the analysis. Only 18 residues (F3, V5, L16, F17, V18, V33, I34, L36, L41, L50, V54, I56, M63, M64, V80, L83, I90, L96) remain as potential prediction targets. The sequence-based method correctly predicted seven (39%), and structure-based method predicted eight (44%) of all targets. Only four (22%) residues were common between the two methods. Eleven (61%) of all targets can be identified by either one of the methods.

As saturated mutagenesis experiments have been performed for CcdB, it enabled us to quantify the number of substitutions at a predicted position that would lead to a temperature sensitive mutant. On average 6.5 substitutions led to Ts mutants at the positions predicted by the structure-based method, as compared to 3.3 substitutions predicted by sequence-based method (Table 3).

**Table 3.** Number of substitutions that led to a Ts mutant at positions identified by different prediction modes in *Escherichia coli* protein CcdB

Prediction method	Number of predictions	Average number of Ts mutants
Exclusively sequence-based	3	3.3
Exclusively structure-based	4	6.5
Sequence-based	7	5.1
Structure-based	8	6.5
Sequence- and structure-based	4	6.5
Sequence- or structure-based	11	5.6

## DISCUSSION and CONCLUSION

Our predictions are based on the assumption that mutating buried hydrophobic residues would destabilize a protein, hence sometimes rendering it temperature sensitive. We have employed two methods, sequence-based and structure-based to make these predictions. The sequence-based method estimates the degree of burial of a residue by the hydrophobicity and hydrophobic moment of it and its neighbouring residues. The structure-based method computes residue depth (extent of burial), a 3D structure or accurate homology model. The aim is to identify a small number of residue positions in proteins and suggests substitutions that are likely to produce Ts mutants. These potential Ts mutants can then be constructed and experimentally screened. Our methods do not attempt to predict either all buried residues or all possible Ts mutants.

We have shown that, both sequence-based and structure-based methods are capable of accurately identifying Ts mutants. The overlap between the two methods is however low. Specifically, the Jaccard index (ratio of the intersection set to the union set) of the sequence-based and the structure-based predictions is only 0.32. This motivates us to suggest that the experimentalists first mutate positions that are common to the two prediction methods. This strategy substantially reduces (halves) the number of predictions and hence increases precision (90–100% in the case study).

In our benchmark set, of 36 experimentally verified Ts mutants, the sequence-based method identified 22 (59%), and the other 15 (41%) were exclusively identified by the structure-based method. Furthermore, none of the structure-based predictions have been invalidated by experiments as yet. However, hydrophobic environments can also be found on protein surface forming active sites and protein-protein interaction interfaces (55). Mutating these non-buried residues could also lead to decreased functional activity of the protein and a Ts mutant. This is shown in the case of *E. coli* CcdB. Both a high-resolution structure and an exhaustive set of Ts mutants is available for the protein. We found that eight Ts mutants correctly predicted with the sequence-based method did not satisfy structure-based burial criteria, and most of these residues are part of the protein active site or are part of a protein-protein interaction interface (23). This suggested that the sequence-based method can be utilized when no structural prediction are forthcoming.

The dependency of structure-based prediction on homology model quality was also investigated. Our results show that structure-based prediction performance was not critically dependent on modeling template as long as they passed the assessment criteria.

As mentioned earlier, the sequence-based method also predicts mutations in positions spatially close to active site and protein-protein interfaces to be temperature sensitive (23). However, our analysis with CcdB reveals that these positions are more selective to mutations as compared to buried positions. It is likely that mutations at positions that are not as deep as core residues are less thermally destabilizing.

Without the availability of large scale and exhaustive studies of Ts mutants, it is not possible to accurately gauge the false positive rate of the method. Hence, a rigorous estimation of sensitivity and specificity of the method is not currently possible. However, for practical purpose, usually one or a few Ts mutants are sufficient to facilitate further investigation of a biological system. Using our testing set, we showed that at least a few high confidence predictions could be made for each case.

Engineering temperature sensitive mutant is a versatile technique that can be used for a variety of purposes as it enables an easy and reversible manipulation of protein function. This server has been set up to provide a simple and intuitive tool to rationally design temperature sensitive mutants. We hope that it will be useful in investigating various biological systems.
